# Analysis of the Nutritional Content, Phytochemicals, and Antioxidant Properties of the Unexploited Wild Grape Variety *Ampelocissus indica* (L.) Planch in Comparison to *Vitis vinifera* L.

**DOI:** 10.1155/ijfo/1582620

**Published:** 2025-08-19

**Authors:** Wathsala Jayani Nandasiri, Isurika Rosini Fernando, Champa Disala Jayaweera

**Affiliations:** Department of Chemistry, Faculty of Applied Sciences, University of Sri Jayewardenepura, Nugegoda, Sri Lanka

**Keywords:** *Ampelocissus indica* (L.) Planch, antioxidant activity, flavonoids, maturity indices, phenolic compounds, proximate analysis

## Abstract

*Ampelocissus indica* (L.) Planch is an unexplored wild grape variety grown in Sri Lanka. This article is aimed at comparing the nutritional, antioxidant, and phytochemical composition of *A. indica* with the commercial variety *Vitis vinifera* to evaluate its potential commercial applications. Both grape varieties were collected at their commercial maturity, and the maturity indices, proximate composition, phytochemicals, and antioxidant activities of the grape samples were analyzed according to modified literature procedures after authentication. Statistical analysis of results was carried out using Dunnett's one-way ANOVA method. Significant differences at a 95% confidence level were observed in *A. indica* samples in carbohydrates, proteins, and fiber contents but not in ash and lipids compared to *V. vinifera*. Qualitative phytochemical analysis indicated the presence of alkaloids, flavonoids, proanthocyanidins, unsaturated sterols, triterpenes, and phenolic compounds in both berries of *A. indica* and *V. vinifera*. Total flavonoid content (TFC), total tannin content (TTC), and total phenolic content (TPC) of *A. indica* samples ranged between 1.36 ± 0.07 and 4.23 ± 1.28 mg quercetin equivalent/100 g of dried weight of plant material (DW), 0.13 ± 0.04–0.26 ± 0.08 mg of tannic acid equivalent/100 g of DW, and 11.48 ± 4.73–29.09 ± 6.05 mg gallic acid equivalent/100 g of DW, respectively. TFC, TTC, and TPC of *A. indica* samples exhibited no statistically significant differences (*p* ≥ 0.05) with those parameters of *V. vinifera*. The antioxidant activity of *A. indica* samples was determined using ferric ion–reducing antioxidant power, and 2,2⁣′-azino-bis(3-ethylbenzothiazoline-6-sulfonic acid) assays exhibited statistical grouping equality (*p* ≥ 0.05) with *V. vinifera*, while 2,2-diphenyl-1-picrylhydrazyl assay did not. The *A. indica* variety, with its significantly low pH values, lower carbohydrate content, and richness in red pigments, can be recommended for use in acidic, low-sugar functional food industry and natural health products in the pharmaceutical industry.

## 1. Introduction

Grape is an economically important food crop widely grown in the subtropical regions of the world [1]. They are classified as commercial and wild varieties based on their habitat or cultivation method. The most cultivated commercial grape variety, *Vitis vinifera* L. is domesticated from wild varieties of grapes [2]. Throughout history, berries of the family *Vitaceae*, especially *V. vinifera*, have been prized for their versatility and flavor [3, 4]. The unique flavor, color, and texture of commercial varieties of grapes have been utilized in manufacturing wines, juices, vinegar, and other edible products. Additionally, berries fulfill the dietary needs of human beings [1, 4].

*Ampelocissus indica*, the “red-stemmed wild grape” [5], is an underutilized plant, native to Southeast Asia [6] and widely grown in the evergreen and semievergreen forests in Sri Lanka [7]. The climbing tubular vine of *A. indica*produces flowers and spherical red berries, annually from March to September. Scientific analysis of secondary metabolites and biological activities of fruits of *A. indica* grown in different parts of the globe is currently unexplored. Proximate composition and qualitative phytochemical analysis of roots of *A. indica* grown in Kerala state in India demonstrated the presence of plant secondary metabolites, including flavonoids, tannins, alkaloids, saponins, and phenolic compounds [8, 9].

Various parts of the *A. indica* plant demonstrate important biological properties including antioxidant, anti-inflammatory, hepatoprotective [8], anticancer, and antidiabetic [10] activities. The roots and berries of this ethnomedicinal plant were used to treat patients with jaundice and liver problems [11] and to treat a variety of ailments, including digestive problems, inflammation, and skin conditions [8, 12] by tribal groups. In addition to the medicinal uses of *A. indica*, the fruits of *A. indica* were utilized as an ingredient in pickling in culinary practices [13]. Despite its ethnomedicinal and ethnobotanical significance, there are no specific records in online databases or scientific literature indicating the worldwide distribution of the exact plant, *A. indica*, beyond South and Southeast Asia, highlighting a gap that underscores the relevance of the present study.

A systematic study on maturity indices, proximate analysis, phytoconstituents, and antioxidant properties of berries of *A. indica* is still lacking except for the preliminary investigation of total phenolic content (TPC) and antioxidant activity of fruits of *A. indica* collected from Kanneliya, Southern Province, Sri Lanka [14]. Among the berries of other species of the *Ampelocissus* genus, phytochemical analysis of *A. latifolia* and *A. martini* has already been reported [15, 16]. Qualitative phytochemical analysis carried out on the methanol extract of fruits of *A. latifolia*, which were collected from Dhaka, Bangladesh, between June 2012 and March 2013, consisted of tannin, flavonoid, saponin, gum, alkaloid, and terpenoids [15–17]. TPC, total flavonoid content (TFC), total proanthocyanidin content, and total saponin content of fruits of *A. martini* exhibited a strong correlation with their antioxidant activity [18].

This research project focused on the systematic and comparative study of maturity indices, proximate analysis, qualitative and quantitative analysis of phytochemicals, evaluation of antioxidant activities, and statistical analysis of fruits of wild grape variety, *A. indica*, grown in three selected geographical locations of Sri Lanka with the fruits of commercial grape variety, *V. vinifera*. This is the first systematic study on proximate analysis and qualitative and quantitative analysis of plant secondary metabolites, along with the evaluation of the antioxidant activity of fruits of *A. indica* grown in Sri Lanka, to the best of our knowledge. Subsequently, the findings of this study provide a fundamental understanding and a proper direction to investigate the potential applications of the underutilized fruits of *A. indica* in the food, pharmaceutical, cosmetics, textile, or related industries.

## 2. Materials and Methods

### 2.1. Chemicals and Instruments

All chemicals, reagents, and solvents were of analytical grade (AR) or American Chemical Society (ACS) grade and used as received without further purification unless otherwise mentioned. Rapid moisture analyzer (Precisa-330 XM), brix meter (Optika, HR-150), texture analyzer (CT3 Texture Analyzer-Brookfield), drying oven (Memmert U30), and freeze dryer (Cool Safe 100-4, Trigon Plus) were used in this project. A UV-visible spectrophotometer (Shimadzu, UV1800) was used in the spectrophotometric assays, and data were processed with UV-WIN software.

### 2.2. Sample Collection, Authentication, Grading, and Storing

Fresh fruits of *V. vinifera* were collected from a grape cultivation located in the geographical coordinates of 9.7501694 N, 79.9538420 E, of Urumpirai in the Jaffna district in the Northern Province of Sri Lanka. Samples of the fresh fruits of the wild variety, *A. indica*, A–D and E–F, were collected from Kanneliya in the Galle district and Siyambalagoda in the Matara district from the Southern Province of Sri Lanka, respectively. The geographical coordinates of Samples A, B, C, and D were 6.15072 N, 80.20383 E; 6.252344 N, 80.347339 E; 6.15069 N, 80.20459 E; and 6.15069 N, 80.20459 E, respectively, while those of Samples E and F were 6.08292 N, 81.29983 E, and 6.05069 N, 80.95329 E, respectively. Six distinct fruit samples were collected from six individual trees located in separate sites within the same ecological region to assess the compositional variability of *A. indica* due to potential geographical and biological influences and to ensure statistical robustness. The fresh fruit samples of *V. vinifera* and *A. indica* were collected at their commercial maturity, 9 weeks after the fully bloomed flowering stage. All samples of *A. indica* were collected in July 2021 and *V. vinifera* samples in September 2021, respectively. The collected fruits were safely transported to the laboratory and stored under refrigerated conditions at 4°C until further use. Plant specimens of *A. indica* and *V. vinifera* were identified to their variety level at the Division of Authentication, Department of National Botanic Gardens, Peradeniya, Central Province, Sri Lanka.

### 2.3. Sample Preparation

The fruits were thoroughly washed with running water. The fruit juice was prepared by chopping the whole fruit with a mortar and pestle and filtering the resulting pulp using filter paper. The oven-dried and freeze-dried powders of fruit samples were prepared separately using the modified literature procedures [19]. The fruits were sliced using a stainless-steel knife and oven dried in a laboratory air-forced oven at ≤ 55°C for 18–24 h. The dried fruits were milled at 5000 rpm for 10 s with a grinder and sieved to obtain particles < 400 *μ*m. Freeze-drying was carried out in a lyophilizer at −40°C (12 Pa) for 48 h. Both oven-dried and freeze-dried samples were stored in tightly sealed double-laminated pouches at < 4°C until further use. The 50% methanol extract of the freeze-dried sample was prepared by macerating the freeze-dried powder of each fruit sample separately in 50% methanol at a room temperature of 30°C for 12 h, followed by filtering. A 50% methanol solution was employed for the extraction of bioactive compounds due to its proven efficiency in solubilizing a wide range of polar phytochemicals. Many of the reported secondary metabolites in grapes and related species, such as phenolics, flavonoids, and tannins, possess multiple polar functional groups such as hydroxyl and carbonyl groups [20], enhancing their solubility in hydroalcoholic systems. The selected solvent composition aligns with AOAC-recommended [20] protocols for laboratory-scale extraction, offering a standardized method that simulates the solubilization potential of biological systems, particularly the human gastrointestinal tract. Thus, this approach ensures the extraction process is both analytically robust and biologically relevant.

### 2.4. Determination of Maturity Indices

The moisture content, pH, titratable acidity (TA), and total soluble solids (TSSs) of freshly prepared fruit juice and the texture profile of fresh fruits of commercial and *A. indica* samples were tested in triplicate according to the modified literature procedures [18].

### 2.5. Proximate Composition Analysis

The ash content, total carbohydrate content, crude protein content, crude fat content, and crude fiber content of oven-dried fruit samples were determined in triplicates using the dry ash method, phenol–sulfuric method, Kjeldahl method, Soxhlet extraction method, and nonenzymatic gravimetric method, by following AOAC procedures only scaling down to accommodate the specific sample sizes, respectively [20]. The results were presented as a percentage on a dry matter basis.

### 2.6. Qualitative and Quantitative Analysis of Phytochemicals

#### 2.6.1. Qualitative Analysis of Phytochemicals

The qualitative analysis of seven selected secondary metabolites in 50% methanol extract of freeze-dried fresh fruit samples was carried out according to the standard procedures [21]. Alkaloids were tested using Wagner's and Mayer's tests while the presence or absence of flavonoids, proanthocyanidins, unsaturated sterols, triterpenes, phenolic compounds, and saponins was determined using the cyanidin test, proanthocyanidin test, Liebermann–Burchard test, Salkowski test, ferric chloride test, and froth test, respectively.

#### 2.6.2. Quantitative Determination of TFC, TPC, and TTC

The TFC, TTC, and TPC of 50% methanol extracts of freeze-dried fruit samples were carried out using the standard spectrophotometric methods, aluminum chloride [22] tannic acid–Fe(III) [23], and Folin–Ciocalteu methods [24] by scaling down to accommodate the specific sample sizes, respectively. Quercetin, tannic acid, and gallic acid were used as the reference compounds for the determination of TFC, TTC, and TPC, respectively. Absorbance measurements for the determination of TFC, TTC, and TPC were recorded at 430, 540, and 765 nm, respectively. Each assay was duplicated.

#### 2.6.3. Determination of Antioxidant Activity

Antioxidant activities of 50% methanol extracts of freeze-dried fruit powders of *V. vinifera* and *A. indica* were separately estimated by carrying out the standard 2,2-diphenyl-1-picrylhydrazyl (DPPH) [25], ferric-reducing antioxidant (FRAP) [26], and 2,2′-azino-bis(3-ethylbenzothiazoline-6-sulfonic acid) (ABTS) [27] spectrophotometric assays using the standard compound, Trolox for the DPPH assay, and ascorbic acid for the FRAP and ABTS assays. Each absorbance measurement for DPPH, FRAP, and ABTS assays was recorded at 517, 593, and 735 nm, respectively. The analyses were triplicated.

### 2.7. Statistical Analysis

Results of maturity indices, proximate analysis, TFC, TTC, TPC, and antioxidant activities of all samples were statistically compared using a statistical method of ANOVA. Moreover, the composition of the above mentioned results of six samples of A, B, C, D, E, and F with the commercial variety sample was performed by following the one-way ANOVA statistical method of Dunnett multiple comparisons, considering 95% confidence levels. *p* ≥ 0.05 implies that the two values belonged to the same group.

In this study, Dunnett's one-way ANOVA test was employed to statistically compare each of the *A. indica* fruit samples collected from different locations against a single commercial *V. vinifera* variety. This method was chosen over other multiple comparison procedures due to its statistical efficiency and its ability to control the family-wise error rate when comparing several treatments to a single standard. Since the primary objective was to identify significant deviations of *A. indica* samples from the reference cultivar across various nutritional, phytochemical, and antioxidant parameters, Dunnett's test offered a direct and scientifically valid comparison framework. Furthermore, this test is especially appropriate in repeated measurement designs where all groups are tested under similar conditions, thus ensuring consistency and reliability of all the separate tests in determining and comparing statistical significance with the commercial *V. vinifera* variety.

## 3. Results and Discussion

This discussion interprets the comparative analysis of proximate composition, phytochemicals, and antioxidant properties between wild *A. indica* and commercial *V. vinifera* grape varieties. The statistical significance of the differences was evaluated to support the findings. The statistical method of one-way ANOVA, the Dunnett test, was used to compare multiple samples with the commercial sample. In this study, the Dunnett test assessed whether there were any significant differences in mean values of the considered parameters of the wild variety and the commercial variety. The *p* value obtained from the Dunnett test is smaller than 0.05, which implies that there is a statistically significant difference between the two varieties.

### 3.1. Determination of Maturity Indices

Maturity indices are preliminary parameters that are used to determine the maturity stage of a natural commodity.

The moisture content of *V. vinifera* fruit juice sample was 79.97% ± 1.43% while that of *A. indica* varied in the range of 80.92% ± 2.91%–87.52% ± 4.84% (Table 1). Among *A. indica* samples analyzed, the highest and lowest moisture contents were observed in Samples E and B, respectively. The moisture content of Samples A and B exhibited *p* values lower than the significance threshold of 0.05 (*p* ≤ 0.05), indicating a statistically significant difference when compared to the commercial variety sample. Conversely, the moisture content of Samples C, D, E, and F displayed grouping equality with the commercial sample. The wet basis moisture content of *V. vinifera* cultivars grown in the Kurdistan region in Iran exhibited the highest moisture content, 84.252%, for the “superior” cultivar, while the lowest moisture content, 73.174%, was for the “Yaghute-Rash” cultivar [28]. In comparison, a study conducted on Thompson seedless grapes at MIT College of Food Technology and ICAR–National Research Centre for Grapes in India reported a moisture content range of 75%–85% in fresh grapes [29]. The values observed in *A. indica* samples in the present study were slightly higher, indicating a comparatively greater moisture retention in this lesser-explored fruit. Additionally, North Caucasian Federal Scientific Center of Horticulture, Viticulture, Wine-making in Russia reported that in other *V. vinifera* cultivars such as Pinot Noir, Saperavi, and Cabernet Sauvignon to have moisture contents ranging from 48.50% ± 0.15% to 55.15% ± 0.91%, while Merlot and Rebo cultivars showed 64.45% ± 0.34% and 64.21% ± 0.62%, respectively [30]. These findings indicate that the *A. indica* samples analyzed in the present study generally possessed a comparatively higher moisture content. These changes in the moisture content in the fruits can be influenced by multiple factors, including varietal characteristics, growing conditions, and postharvest processing [31]. The *A. indica* samples in this study were grown in wetland regions of Sri Lanka and processed with minimal delay, ensuring sample freshness and preventing moisture loss due to wilting. These conditions may have contributed to the comparatively higher observed moisture retention.

*V. vinifera* fruit juice samples demonstrated an acidic pH of 3.08 ± 0.45. Among *A. indica* samples analyzed, Sample F demonstrated the highest pH value of 2.83 ± 0.27 while the lowest pH value of 2.62 ± 0.26 was demonstrated by Sample B (Table 1). Grape juice exhibited acidic pH, typically having a pH spanning from 3.2 to 4.0 [31]. During the harvesting period, malic acid and tartaric acid are responsible for more than 90% of the acidic content in grapes [31]. Out of the six *A. indica* samples analyzed, pH values of only D and F samples demonstrated (*p* > 0.05), suggesting no statistically significant difference in pH when compared to the commercial group, and thus, they were categorized within the same statistical group. The grape pomace derived from the Öküzgözü variety and sourced from a commercial winery in the Çal district of Denizli, Turkey, during the 2016 vintage, exhibited a pH value of 3.33, which is slightly higher than the values observed in *A. indica* samples, indicating a relatively stronger acidic nature in *A. indica* fruits [31]. These differences in pH may primarily stem from the inherent varietal characteristics of *A. indica*, which is known to possess naturally higher levels of organic acids compared to common *V. vinifera* varieties like Öküzgözü. Additionally, environmental [32] and agronomic factors, such as *A. indica*'s cultivation in the humid, wetland regions of Sri Lanka, may favor the accumulation of organic acids. The careful handling and immediate processing of the freshly harvested *A. indica* fruits further helped retain their native acid profile [31] by preventing enzymatic degradation or microbial fermentation. In contrast, grape pomace from the Öküzgözü variety, being a by-product of winemaking, may undergo partial acid loss during processing or storage, contributing to its slightly higher pH value [31–33].

The TA of *V. vinifera* was 9.43 ± 1.18 g/L while that of the fruit samples of *A. indica* varied in the range of 8.13 ± 0.65–13.11 ± 0.99 g/L. Among *A. indica* samples analyzed, the highest TA was observed in Sample A and the lowest TA was observed in Sample D (Table 1). The usual range for TA found in grapes was between 5.0 and 16.0 g/L expressed as tartaric acid equivalent [33]. When considering the TA content, Samples D, E, and F showed no statistically significant difference (*p* ≥ 0.05), indicating that there is no difference with the commercial *V. vinifera* sample. In contrast, the TA of Samples A–C demonstrated statistically significant differences (*p* ≤ 0.05) from the commercial sample. In comparison, the TA of unripen fresh grape pomace from the Öküzgözü variety of *V. vinifera*, which was sourced from a commercial local winery in the Çal district of Denizli, Turkey, during the 2016 vintage, was recorded at 4.38% [31], equivalent to approximately 43.8 g/L, indicating a markedly higher acidity than that observed in whole grape of *A. indica* fruit samples.

The TA of *V. vinifera* in the present study was 9.43 ± 1.18 g/L, while that of *A. indica* ranged from 8.13 ± 0.65 to 13.11 ± 0.99 g/L, aligning with the typical TA range of 5.0–16.0 g/L reported for grapes. Variations in TA values among the samples can be influenced by factors such as fruit maturity, cultivar differences, and environmental conditions [34]. For instance, the significantly higher TA, 43.8 g/L, reported in unripe grape pomace of the Öküzgözü variety [31] may be attributed to the early harvest stage and concentration of acids in the pomace [34], which differ chemically from whole fruit. Additionally, statistically significant differences (*p* ≤ 0.05) among Samples A–C compared to the commercial grape suggest varietal or compositional distinctions in organic acid content.

The TSS value of the commercial variety, *V. vinifera*, was 10.83 ± 3.93°Brix. Among the *A. indica* samples, the highest TSS value of 13.25 ± 1.70°Brix was exhibited by Sample F, while the lowest TSS value of 5.25 ± 1.61°Brix was shown by Sample D (Table 1). The TSS values of Samples E and F were not significantly different (*p* ≥ 0.05) from that of the commercial *V. vinifera* sample, indicating statistical grouping similarity, whereas the remaining samples exhibited significant differences. The TSS of unripe *V. vinifera* collected from vineyard plots in the Cal district, Denizli, ranged from 4.1 to 6°Brix [31]. Another study reported the TSS values of 15.7°Brix in Swenson Red and 20.8°Brix in Frontenac of matured fruits of *Vitis* sp., which were grown in a vineyard located near Krakow in Poland [35]. In another related study, the TSS content of fully matured fresh grapes harvested in August 2018 from the Ampelos grape cultivation station in Vrbovec, Czech Republic, ranged from 13.33 ± 1.45 to 15.67 ± 0.66°Brix among the *V. vinifera* varieties Jakubské, Beauty Seedless, Vrboska, Bezsemenné, and Perlette, highlighting the variability of TSS levels across different cultivars and growing conditions. In comparison, the TSS values of the grape varieties in a study conducted in Vrbovec, Czech Republic, ranged from 13.33 ± 1.45 to 15.67 ± 0.66°Brix, which were generally higher than the TSS value of the commercial *V. vinifera* sample, 10.83 ± 3.93°Brix [36], and most *A. indica* fruit samples analyzed in the present study. The reported higher TSS values may be attributed to several factors, including the use of high-sugar accumulating cultivars, optimal vineyard management, extended ripening periods under favorable climatic conditions, and lower water availability, which can enhance sugar concentration through mild water stress. In contrast, the comparatively lower TSS value observed in the commercial *V. vinifera* sample in the present study could be due to early harvesting, varietal characteristics with inherently lower sugar accumulation, less favorable environmental conditions, or postharvest handling variations. These findings underscore the significant influence of genotype [36], ripeness stage, and environmental and agronomic practices on the soluble solid content of grape fruits [34, 35].

Surface hardness, resilience, cohesiveness, gumminess, springiness, and chewiness were determined as texture profile parameters. The surface hardness of fruits of *A. indica* varied from 1.55 ± 0.47 to 19.38 ± 13.05 N, while that of *V. vinifera* showed 2.86 ± 2.49 N. The highest and lowest surface hardness values were demonstrated by Samples E and F, respectively (Table 2). The hardness of grape berries of Kozma Palne Muscat and Melinda varieties in the Matra wine region, Hungary, was 0.787 and 1.209 N, respectively [37]. Another study reported that the skin break force of three red grape varieties, namely, Mencía, Brancellao, and Merenzao, grown in Galician vineyards in Northwest Spain varied between 0.521 and 0.811 N [38]. The current study showed higher surface hardness values than Mencía, Brancellao, and Merenzao grape varieties.

The resilience across the fruits of *A. indica* displayed a range from 0.29 ± 0.04 to 0.38 ± 0.07, whereas that of *V. vinifera* fruits was 0.49 ± 0.06. In the *A. indica* samples, the highest level of resilience of 0.38 ± 0.07 was observed in Sample E, while the lowest level of resilience of 0.29 ± 0.40 was evidenced in Sample D (Table 2). A study has reported that the resilience values of two grape varieties, Melinda and Kozma Pálné Muscat in the family *Vitaceae* which were grown in the Matra wine region, Hungary, were 0.227 and 0.209, respectively [37], which were higher than those of the current study.

Among the *A. indica* samples, the highest cohesiveness value of 0.65 ± 0.16 was observed in Sample F, and the lowest cohesiveness value of 0.54 ± 0.10 was observed in Sample D (Table 2). *V. vinifera* fruits showed cohesiveness of 0.85 ± 0.38. According to research on berries grown in Hungary, the cohesiveness values of grape berry varieties, Melinda and Kozma Pálné Muscat, were 0.498 and 0.466, respectively [37], which were lower than the cohesiveness values observed in the present study.

The gumminess spanned from 102.47 ± 36.05 to 1158.00 ± 824.00 N in the fruits of *A. indica*, and the *V. vinifera* sample showed the gumminess of 229.60 ± 158.70 N. Among *A. indica* samples analyzed, the highest and lowest gumminess values were indicated by Samples E and D, respectively (Table 2). A research study on the Melinda and Kozma Pálné Muscat grape varieties belonging to the family *Vitaceae* showed the gumminess of 0.498 and 0.466 N, respectively [37]. These gumminess values reported were lower than those of the present study.

In the fruits of *A. indica*, the springiness values ranged from 1.40 ± 0.14 to 2.51 ± 2.34 mm, whereas for *V. vinifera*, the springiness values observed were 2.29 ± 1.86 mm. Sample E showed the highest value, while Sample D showed the lowest value for the springiness of the analyzed samples of *A. indica* (Table 2). As per a research study, the springiness of Melinda and Kozma Pálné Muscat grape berries was 3.665 and 4.042 mm [37] which were higher than the springiness values of the current study.

The chewiness value of the fruits of *A. indica* ranged from 1.44 ± 0.44 to 18.85 ± 12.61 mJ, while that of *V. vinifera* was 3.94 ± 2.48 mJ. Sample C showed the highest chewiness value, and Sample F exhibited the lowest chewiness value among the analyzed *A. indica* samples (Table 2). In a recent research study, the chewiness values of two varieties in the grape family were reported as 1466 and 2216 mJ for the Melinda and Kozma Pálné Muscat grape varieties [37] which were higher than the values observed in this study.

In the current study, maturity indices of six samples of *A. indica* were compared against the commercial variety, *V. vinifera*, with a focus on texture attributes such as hardness, resilience, cohesiveness, gumminess, springiness, and chewiness employed in texture profile analysis. The hardness of Samples A and F showed (*p* ≥ 0.05), indicating their grouping equality of hardness. Conversely, the hardness of Samples B, C, D, and E differed significantly from that of the commercial sample. For resilience and cohesiveness, all samples exhibited low *p* values compared to the commercial sample, suggesting consistency in these attributes across the tested samples. In the case of gumminess, Samples A and F had notably higher *p* values, indicating their grouping equality in gumminess, while Samples B, C, D, and E showed significant differences from the commercial sample in this aspect. Springiness showed significant variations for all samples, with each sample displaying distinct springiness characteristics compared to the springiness of the commercial sample. Chewiness varied significantly, with Samples A, D, and F exhibiting higher *p* values, indicating similar grouping in chewiness profile compared to the commercial sample, while Samples B, C, and E differed significantly in chewiness from the commercial sample.

The variations observed in texture profile parameters such as surface hardness, resilience, cohesiveness, gumminess, springiness, and chewiness between the present study and previously reported values can be attributed to differences in fruit type, cultivar-specific characteristics, and ripeness at the time of harvest. *A. indica* and *V. vinifera* possess distinct structural properties, while environmental factors [34, 35], growing conditions, and postharvest handling can further influence textural attributes [37]. Additionally, methodological differences in texture analysis protocols may also contribute to the observed discrepancies [37, 38].

### 3.2. Proximate Composition Analysis

When preparing the oven-dried powdered fruit samples for proximate composition analysis, the moisture content was brought below 5% on a dry matter basis to ensure the long-term stability of powdered samples and to prevent fungal attacks during storage. The targeted moisture content was achieved by extending the drying duration, followed by assessing the moisture content with a rapid moisture analyzer.

Among the *A. indica* fruits analyzed, Sample B demonstrated the highest ash content of 0.07% ± 0.00%, while the commercial sample and Samples E and F demonstrated the lowest ash content of 0.04% ± 0.00% on a dry matter basis (Table 3). When statistically examining the ash content, Samples D, E, and F were equivalent to the commercial sample, as indicated by the Dunnett grouping with higher *p* values (*p* ≥ 0.05). A research study of the ash content of *V. vinifera* collected from Grombalia in the northeast of Tunisia was 4.50% ± 0.05% [39]. Another study reported the ash content as 3.15% on a dry weight basis of *V. vinifera* grapes, which were collected from Explotaciones Hermanos Delgado organic winery in Spain [40]. These reported ash contents were higher than the ash contents of both varieties analyzed in our study [39, 40].

In the present study, both *A. indica* and *V. vinifera* samples showed a lower ash content ranging from 0.04% to 0.07%, which is significantly lower than the reported 3.15%–4.50% for *V. vinifera* in Tunisia. These discrepancies could result from differences in location, soil mineral composition, or fruit developmental stages [35]. Additionally, the precise drying protocol used in this study, which reduced moisture to below 5%, might have influenced the retention or detectability of mineral residues and methodological slight variations [39] in ash determination could also account for these lower values.

Among the analyzed A to F samples of *A. indica*, the highest carbohydrate content of 56.21% ± 5.43% was exhibited by Sample F, and it was lower than that of the commercial sample, which was 56.97% ± 13.73%. The lowest carbohydrate content of 37.39% ± 10.03% was obtained in Sample A (Table 3). In the analysis of proximate composition, it was found that only the E and F samples did not exhibit statistically significant differences (*p* ≤ 0.05) in terms of carbohydrate content. A research study of Cabernet Sauvignon and Sauvignon Blanc grape varieties collected from Adelaide, Australia, reported the carbohydrate content as 31%–54% on a dry weight basis [41]. These values aligned with the values observed in the present study. A study on the nutritional composition of *V. vinifera* collected from Grombalia in Northeast Tunisia reported a carbohydrate content of 82.50 ± 0.00 g/100 g, which was obtained by subtracting the total sum of protein, fat, ash, and moisture from a 100% dry weight sample [39]. The carbohydrate content of *V. vinifera* berries reported [38] was higher than that of the reporting study. The proximate composition of red grape pomace collected from a Faragello factory in Egypt reported that the total carbohydrate percentage was 74.2%, which was determined by following the same weight differential method [39, 41].

Carbohydrate content in *A. indica* ranged from 37.39% to 56.21%, while the commercial *V. vinifera* sample showed 56.97%, aligning well with the 31%–54% range reported for grape varieties like Cabernet Sauvignon [41]. However, higher carbohydrate values such as 82.5% reported in Tunisian *V. vinifera* [38] may reflect overestimations from weight differential calculations or differences in sample composition. Factors such as ripening stage, variety [36], and regional climate [35] could significantly affect carbohydrate accumulation. Analytical method differences also play a key role in reported variability across studies [39].

Among all the samples analyzed, Samples A and E exhibited the highest and lowest fat content of 4.90% ± 0.72% and 2.73% ± 0.32%, respectively, while *V. vinifera* demonstrated a fat content of 4.74% ± 2.88% (Table 3). The fat content of *V. vinifera* fruits, which were collected from Grombalia in the northeast of Tunisia, was 2.87 ± 0.02 g/100 g [39]. A separate investigation reported that red grape pomace contains a fat content of 11.5% on a dry weight basis. None of the samples in this current study exhibited a higher fat content than that of the red grape pomace [4]. Furthermore, when examining the fat content of D, E, and F samples, they were equivalent to the commercial sample, as indicated by the Dunnett grouping with higher *p* values (*p* ≥ 0.05).

The fat content in *A. indica*, 2.73%–4.90%, and *V. vinifera*, 4.74%, fell below that of red grape pomace, 11.5%, likely due to the higher lipid concentration in grape seeds and skins found in pomace. Since the current study focused on whole fruits, the fat levels were naturally lower. Lipid biosynthesis can be influenced by environmental stresses [35] and varietal traits [32], but in general, fleshy fruits like grapes are low in fat [32]. Extraction technique and sample preparation also significantly affect the quantified fat content [30, 32].

The commercial variety, *V. vinifera*, demonstrated a protein content of 7.22% ± 1.21%. The protein contents of *A. indica* fruits varied in the range of 3.65% ± 1.10%–6.80% ± 0.10%, where the highest and lowest protein contents were shown by Samples C and A, respectively (Table 3). The protein content of *V. vinifera* fruit was reported in two separate studies as 6.93 ± 0.01 g/100 g [35] and 11.8 ± 0.08 g/100 g [38] on a dry matter basis. The protein content of red grape pomace, which was collected from a Faragello factory in Egypt, was reported as 12.5% in a study carried out [38]. Considering the protein content, only Sample C did not exhibit a statistically significant difference from the commercial variety, whereas all other samples showed significant differences (*p* ≤ 0.05).

The protein content of *A. indica* fruits ranged from 3.65% to 6.80%, and *V. vinifera* demonstrated 7.22% ± 1.21%, which corresponds with previously reported grape values around 6.93% but remains lower than protein-rich red grape pomace, 12.5%. These differences are likely due to the tissue type analyzed, whole fruit versus concentrated seed or skin fractions in pomace. Genetic background, soil nitrogen availability, and postharvest processing could further influence protein content [32]. Drying and extraction methods may also impact measurable protein levels [32].

The highest fiber content of 12.42% ± 0.80% was observed in Sample A of *A. indica*, and this value was higher than 9.75% ± 1.31% obtained for the commercial variety, *V. vinifera*. The lowest fiber content of 9.46% ± 0.66% was demonstrated by Sample D (Table 3). However, when assessing the fiber content, it was observed that all samples, except Sample A, displayed similar statistical grouping. This suggests that Sample A exhibited a distinct fiber composition with a lower *p* value (*p* ≤ 0.05) compared to the commercial sample. The crude fiber content of *V. vinifera* fruits, which were collected from Explotaciones Hermanos Delgado organic winery in Spain, was 14.6% ± 0.16% [40]. The red grape pomace obtained from the Faragello factory in Egypt was reported to be 9.2% fiber on a dry weight basis [42].

The fiber content in *A. indica* ranged from 9.46% to 12.42%, and *V. vinifera* showed 9.75%, both slightly lower than the 14.6% reported for Spanish grape varieties. The observed variations in this richer fiber content among the samples are nutritionally significant, as higher dietary fiber intake is associated with improved gastrointestinal health, better glycemic control, and reduced risk of chronic diseases by delaying absorption of carbohydrates in the gastrointestinal tract [8]. Environmental conditions [35], cultivar-specific traits [36], and sample composition play essential roles in determining fiber content. Furthermore, analytical differences, such as the grinding and sieving process, may also influence the fiber values observed in this and other studies [32].

### 3.3. Qualitative and Quantitative Analysis of Phytochemicals

#### 3.3.1. Qualitative Analysis of Phytochemicals

The qualitative analysis of alkaloids, flavonoids, proanthocyanidins, unsaturated sterols, triterpenes, phenolic compounds, and saponins was carried out to obtain a comparison of the above mentioned phytoconstituents in the grape samples (Table 4). The yellow and reddish–brown precipitates resulted from Mayer's and Wagner's tests, respectively, indicating the presence of alkaloids in each sample. The formation of red-color solutions for both cyanidin and proanthocyanidins tests indicated the presence of flavonoids and proanthocyanidins in each sample. The Liebermann–Burchard test indicated blue–green-color solutions with similar intensities for all the analyzed samples, indicating the presence of sterols. The Salkowski test demonstrated a cherry-red color interface between the organic and aqueous layers with similar intensities exhibiting the presence of terpenoids in each sample. The intensity of the deep purple color as indicated by the ferric chloride test suggested the presence of phenolic compounds in all the analyzed samples. The formation of a honeycomb froth of about 4 cm was not observed in the analyzed samples (Table 4) indicating the absence of saponins.

The diverse groups of naturally occurring compounds present in *A. indica*, including alkaloids, flavonoids, tannins, and polyphenolics, are known for their wide-ranging biological activities, including analgesic, antimicrobial, and anti-inflammatory properties, making them valuable in therapeutic applications for managing pain, infections, and chronic inflammatory conditions [15, 17].

#### 3.3.2. Quantitative Determination of TFC, TPC, and TTC

The TFC of 50% methanol extract of freeze-dried fruit powders was determined using the calibration curve with an *R*^2^ of 0.9928; *y* = 76.363*x* − 0.0043, and the TFC values were expressed as milligram of quercetin equivalents (QE) per 100 g of dried weight (mg QE/100 g DW). Among the seven samples analyzed, including *V. vinifera* and *A. indica*, the highest TFC of 4.23 ± 1.28 mg QE/100 g DW was exhibited by Sample E, while the lowest content of 1.36 ± 0.07 mg QE/100 g DW of TFC was observed in Sample A (Table 5). The TFC of *V. vinifera* samples collected at the ripening stage from the *Vitis* clonal repository in Davis, California, of the United States Department of Agriculture-Agricultural Research Service (USDA-ARS) exhibited a wide variation, ranging from 94.7 to 1055 mg QE/100 g on a wet basis [43]. In another study, *V. vinifera* fruits purchased during their maturation period from the local market of Constantine in Northeast Algeria exhibited a TFC of 14.37 ± 0.04 mg QE/g DW [44], which was notably higher than the 3.50 ± 2.11 mg QE/100 g DW observed in the present study. This notable difference in TFC values may be due to genetic variability among grape cultivars [36], as well as differences in postharvest handling, drying methods, and storage duration, all of which can influence the retention and stability of flavonoid compounds during sample preparation and analysis.

Flavonoids, widely studied for their antioxidant and anti-inflammatory effects, can scavenge free radicals and modulate cell signaling pathways. These properties contribute to cardiovascular protection, immune modulation, and chronic disease prevention, thereby supporting the potential application of *A. indica* in nutraceutical and therapeutic formulations [8, 15].

The TTC of 50% methanol extract of freeze-dried fruit powders was determined using the calibration curve with *R*^2^ of 0.9945; *y* = 286.54*x* − 0.0018, and the TTC was expressed as milligram of tannic acid equivalents (TAE) per 100 g of dried weight (mg TAE/100 g DW) of the plant material. Among all the samples analyzed, the highest TTC of 0.26 ± 0.08 TAE/100 g DW was demonstrated by Sample A, and the lowest TTC of 0.17 ± 0.02 mg TAE/100 g DW was observed in Sample B (Table 5). In another study, *V. vinifera* varieties procured from Chriha, Raseki, Assli, and Meski regions of Tunisia were evaluated for TTC using the same tannic acid–Fe^3+^ assay, reporting a range of TTC of 189.03 ± 0.5–208.6 ± 0.4 mg catechin equivalents per gram of dry weight [45]. However, this TTC was not comparable with this reporting study because of the differences in the standard compounds used [45]. This significant discrepancy in TTC values is primarily due to the use of different standard compounds for quantification. The use of tannic acid in the present study versus catechin in the referenced study affects the expression and comparability of TTC results, as different standards have distinct molecular weights and colorimetric reactivity in the assay.

In terms of health, these tannins contribute to gastrointestinal health by modulating gut microbiota, reducing inflammation, and improving mucosal integrity [15]. Additionally, tannins have been reported to exhibit anticancer and antidiabetic potential by influencing cellular pathways related to oxidative stress and metabolic regulation [8]. Their astringent nature also aids in wound healing and oral hygiene, making them valuable in both food preservation and pharmaceutical applications [17].

The TPC of 50% methanol extract of freeze-dried fruit powders was determined using the calibration curve with *R*^2^ of 0.9877; *y* = 4.5405*x* + 0.0874, and the TPC values were expressed as milligram gallic acid equivalents (GAE) per 100 g of dried weight (mg GAE/100 g DW) of plant material. The TPC of *V. vinifera* was 21.17 ± 8.62 mg GAE/100 g DW. The highest TPC of 29.09 ± 6.05 mg GAE/100 g DW was observed in Sample C of *A. indica* which was higher than that of the commercial variety sample. The lowest TPC of 11.48 ± 4.73 mg GAE/100 g DW was observed in Sample A (Table 5). The phenolic profiles of 24 selected *V. vinifera* grape cultivars collected from the USDA-ARS *Vitis* clonal repository in Davis, California, which were determined by following the same method and standard were varied in a wide range of 95.3–686.5 mg GAE/100 g on a wet basis [43]. Another study on the TPC of 22 grape varieties of *V. vinifera* cultivars grown in the Marmara region of Turkey ranged from 9.26 to 167.42 mg GAE/100 g fresh weight of grapes [46]. The TPC studies reported on a fresh weight basis and those TPC values were not comparable with the TPC of the reporting study [45, 46]. A study reported the TPC content of *V. vinifera* grapes from a local market of Constantine in Northeast Algeria, as 23.06 ± 0.17 mg GAE/g DW [44]. The wide variation in TPC among different studies can also be attributed to genotypic differences among grape varieties, as phenolic biosynthesis is highly influenced by cultivar-specific genetic expression [46]. Environmental factors, such as altitude, temperature, sunlight exposure, and soil composition, also play critical roles in determining phenolic accumulation in grape berries [47]. For instance, grapes grown in Mediterranean climates typically exhibit higher phenolic content due to increased sunlight and temperature during ripening [48]. Agronomic practices, including irrigation frequency, pesticide use, and harvesting time, may further impact phenolic content [49]. Moreover, postharvest processing and extraction conditions, such as drying method, solvent concentration, extraction temperature, and duration, greatly affect phenolic yield [50]. Methanol–water mixtures are commonly used; however, variations in polarity and solvent strength can influence the efficiency of extracting specific phenolic subclasses [51]. For example, a study of the grapes from the local market of Constantine in Northeast Algeria [44] reported a much higher TPC of 23.06 ± 0.17 mg GAE/g DW in *V. vinifera*, likely due to differences in extraction parameters and sample preparation techniques.

Phenolic compounds are renowned for their potent antioxidant properties, which help neutralize free radicals and reduce oxidative stress [15], thereby lowering the risk of chronic diseases such as cardiovascular disease, diabetes, and cancer [15]. They also exhibit anti-inflammatory, antimicrobial, and neuroprotective effects [8, 15, 47].

In terms of TFC, TPC, and TTC, all the samples of *A. indica*, A, B, C, D, E, and F, showed *p* values higher than 0.05, indicating none of the samples are statistically significantly different (*p* ≥ 0.05) from the commercial sample. Dunnett grouping information, with 95% confidence, in multiple comparisons, revealed similar grouping in the analysis of TFC, TPC, and TTC when each test was compared to the commercial sample.

The TPC of each sample analyzed in the current study was higher than the sum of the TFC and TTC of the corresponding sample because phenolics encompass a broader spectrum of compounds, including flavonoids and tannins.

#### 3.3.3. Determination of Antioxidant Activity

The antioxidant activities of *V. vinifera* and *A. indica* were evaluated using DPPH, ABTS, and FRAP spectrophotometric assays. The antioxidant activity by the DPPH, ABTS, or FRAP scavenging method is often reported as half-maximal inhibitory concentration (IC_50_), which is defined as the concentration of the antioxidant needed to inhibit the initial DPPH, ABTS, or FRAP concentration by 50% (Table 5).

Trolox, the standard of DPPH assay, demonstrated an IC_50_ value of 8.80 ± 0.66* μ*g/mL. Among the *A. indica* samples analyzed, the highest IC_50_ value of 63.30 ± 17.32* μ*g/mL exhibited by Sample A demonstrated the lowest antioxidant activity, while the lowest IC_50_ value of 17.38 ± 5.48* μ*g/mL shown by Sample D gave the highest antioxidant activity. *V. vinifera* sample demonstrated an IC_50_ value of 24.26 ± 10.75* μ*g/mL, which is lower than the antioxidant activity of Sample D (Table 5). In a previously conducted research study, the antioxidant content of *V. vinifera* fruits was investigated following DPPH assay, and the antioxidant activity was reported as an IC_50_ value of 0.270 ± 0.001 mg/mL [52]. The reported IC_50_ value was lower than the calculated values in the current study. In another related study, red and white grapes collected in Conegliano Veneto and Asti, Italy, in the Mediterranean area showed antioxidant activity in between 4.88 ± 0.18 and 0.32 ± 0.001 mg/g [48] much lower than the present study. Seedless table grape berries of six *V. vinifera* varieties, Autumn Crisp and Pristine (white), Scarlotta and Crimson (red), and Adora and Melody (black)—cultivated in Murcia, Spain, and supplied by SAT MOYCA (Murcia), were collected at their optimum maturity stage and evaluated for antioxidant activity using the DPPH radical scavenging assay. The IC_50_ values for these grape samples ranged approximately from 3.69 to 14 mg/mL on a fresh weight basis [49], indicating comparatively lower antioxidant activity than that observed in the present study. These higher IC_50_ values may be attributed to differences in grape variety [46], geographical origin, growing conditions [46], or extraction protocols [50]; for example, a previous study reported significantly lower IC_50_ values ranging from 0.32 ± 0.001 to 4.88 ± 0.18 mg/g in red and white grapes collected in Conegliano Veneto and Asti, Italy [48], reflecting the influence of environmental factors [47] on antioxidant composition.

In the ABTS assay, the standard ascorbic acid demonstrated an IC_50_ value of 6.38 ± 0.00* μ*g/mL. Among the samples analyzed, the highest IC_50_ value of 79.60 ± 34.20* μ*g/mL was obtained for Sample F, which gave the lowest antioxidant activity, and the lowest IC_50_ value of 58.20 ± 29.40* μ*g/mL was obtained for the commercial sample, which gave the highest antioxidant power. In a previously conducted research study, the IC_50_ value was reported as 0.040 ± 0.003 mg/mL [52], lower than the corresponding value obtained in the present study. This discrepancy may be attributed to differences in grape cultivar [46], environmental growing conditions [47], or the extraction method and solvent polarity, all of which can significantly influence the concentration and activity of antioxidant compounds measured by the ABTS assay.

In the FRAP assay, ascorbic acid standard demonstrated an IC_50_ value of 5.28 ± 0.00* μ*g/mL. The highest value of IC_50_, 66.50 ± 21.7* μ*g/mL, was obtained for Sample D which gave the lowest antioxidant activity, and the lowest IC_50_ value of 44.88 ± 6.20* μ*g/mL obtained for Sample B gave the highest antioxidant power compared to the ascorbic acid standard. *A. indica* samples showed a range between 44.88 ± 6.20-66.50 ± 21.7* μ*g/mL while *V. vinifera* demonstrated an IC_50_ value of 24.26 ± 10.75* μ*g/mL (Table 5). In another study, the antioxidant activity measured by the FRAP assay showed an IC_50_ value of 0.98 ± 0.01 mg/mL [52], which was lower than the value obtained in the present study. This variation may be due to differences in the phenolic composition and redox potential of the samples, as the FRAP assay specifically measures the reducing power of antioxidants rather than their radical scavenging capacity [48, 49].

Based on the Dunnett test results, Samples A and C differ significantly from the commercial sample in terms of antioxidant content following the DPPH assay, while Samples B, D, E, and F exhibit no significant differences (*p* ≤ 0.05) from the commercial sample. Samples A, B, C, D, E, and F indicated grouping equality with the commercial sample for antioxidant content, considering both ABTS and FRAP assays. The above-explained antioxidant profile of *A. indica* samples demonstrated comparable activity to the commercial *V. vinifera* variety in ABTS and FRAP assays, while selected samples showed statistically significant variation in DPPH radical scavenging capacity, reflecting subtle differences in their phytochemical composition, compared to the commercial variety, *V. vinifera*.

## 4. Conclusions

This study provides information and a fundamental understanding of maturity indices, proximate composition, phytochemicals, TFC, TTC, TPC, and antioxidant activities of the samples of wild variety *A. indica* and the commercial variety *V. vinifera* grown in Sri Lanka. Statistical analysis of maturity indices, TA, protein content, and some textural parameters such as resilience and springiness of two grape varieties demonstrated no statistical variation of maturity between the samples. Furthermore, the ash and fat contents of the berries of the wild variety showed statistically equal grouping, whereas carbohydrates, proteins, and fiber were not in the same statistical group of the commercial variety. Qualitative phytochemical analysis indicated the presence of alkaloids, flavonoids, proanthocyanidins, unsaturated sterols, triterpenes, and phenolic compounds in both berries of *A. indica* and *V. vinifera*. TFC, TPC, and TTC of *A. indica* samples exhibited no statistically significant differences with those parameters of the commercial sample. Antioxidant activity of *A. indica* samples determined using FRAP and ABTS assays exhibited statistical grouping equality with the commercial sample, while the DPPH assay did not. The findings of this study, the significantly low pH values, lower carbohydrate content, and richness in red pigments in the berries of the *A. indica* variety can direct the next phase of research to investigate the potential applications of underutilized fruits of *A. indica* to be used in the functional food industry. These findings further highlight the potential of *A. indica* as a valuable alternative fruit source with promising industrial applications in the pharmaceutical sectors apart from food and as a functional ingredient, owing to its favorable physicochemical, textural, and nutritional properties. Future investigations should focus on assessing the bioavailability and metabolic fate of bioactive compounds present in *A. indica*, as well as conducting comprehensive sensory analyses in functional food prototypes to evaluate their technological applicability and consumer acceptance.

## Figures and Tables

**Table 1:** Moisture percentage, pH value, titratable acidity, and total soluble solids of *V. vinifera* and *A. indica*.

**Sample**	**Moisture%**	**pH value**	**TA (g/L)**	**TSS (°Brix)**
ComV	79.97 ± 1.43^a^	3.08 ± 0.45^a^	9.43 ± 1.18^a^	10.83 ± 3.93^a^
A	85.63 ± 4.85	2.65 ± 0.26	13.11 ± 0.99	5.58 ± 1.91
B	87.52 ± 4.84	2.62 ± 0.26	13.06 ± 1.22	6.17 ± 2.38
C	84.28 ± 2.81^a^	2.64 ± 0.18	12.56 ± 0.63	5.92 ± 2.01
D	83.80 ± 2.36^a^	2.73 ± 0.18^a^	8.13 ± 0.65^a^	5.25 ± 1.61
E	80.92 ± 2.91^a^	2.65 ± 0.21	8.63 ± 0.26^a^	8.00 ± 3.03^a^
F	82.21 ± 3.78^a^	2.83 ± 0.27^a^	9.71 ± 0.83^a^	13.25 ± 1.70^a^

*Note:* The values were expressed as mean ± SD of replicates.

Abbreviation: ComV, the commercial variety (*V. vinifera*).

^a^The statistical grouping of Dunnett test.

**Table 2:** Texture profile of *V. vinifera* and *A. indica*.

**Sample**	**Hardness (N)**	**Resilience**	**Cohesiveness**	**Gumminess (N)**	**Springiness (mm)**	**Chewiness (mJ)**
ComV	2.86 ± 2.49^a^	0.49 ± 0.06^a^	0.85 ± 0.38^a^	229.60 ± 158.70^a^	2.29 ± 1.86^a^	3.94 ± 2.48^a^
A	3.37 ± 1.40^a^	0.37 ± 0.05	0.62 ± 0.11	455.00 ± 332.10^a^	1.52 ± 0.13^a^	6.40 ± 4.21^a^
B	18.61 ± 7.78	0.31 ± 0.06	0.56 ± 0.11	981.90 ± 491.50	1.54 ± 0.26^a^	15.45 ± 9.55
C	19.09 ± 8.78	0.37 ± 0.03	0.59 ± 0.09	1113.00 ± 597.00	2.05 ± 1.64^a^	18.85 ± 12.61
D	10.94 ± 8.33	0.29 ± 0.04	0.54 ± 0.10	625.00 ± 567.00	1.40 ± 0.14^a^	4.93 ± 3.96^a^
E	19.38 ± 13.05	0.38 ± 0.07	0.64 ± 0.13	1158.00 ± 824.00	2.51 ± 2.34^a^	18.32 ± 15.28
F	1.55 ± 0.47^a^	0.38 ± 0.05	0.65 ± 0.16	102.47 ± 36.05^a^	1.58 ± 0.12^a^	1.44 ± 0.44^a^

*Note:* The values were expressed as mean ± SD of replicates.

Abbreviation: ComV, commercial variety (*V. vinifera*).

^a^Statistical grouping of Dunnett test.

**Table 3:** The percentages of moisture, ash, protein, carbohydrate, fat, and fiber contents of *V. vinifera* and *A. indica* on a dry matter basis.

**Sample**	**Moisture%**	**Ash%**	**Carbohydrate%**	**Fat%**	**Protein%**	**Fiber%**
ComV	3.12 ± 0.19	0.04 ± 0.00^a^	56.97 ± 13.73^a^	4.74 ± 2.88^a^	7.22 ± 1.21^a^	9.75 ± 1.31^a^
A	5.84 ± 0.16	0.06 ± 0.02	37.39 ± 10.03	4.90 ± 0.72^a^	3.65 ± 1.10	12.42 ± 0.80
B	3.59 ± 1.41	0.07 ± 0.00	44.06 ± 6.55	4.23 ± 0.78^a^	5.64 ± 0.55	9.98 ± 1.17^a^
C	4.20 ± 0.77	0.06 ± 0.00	41.02 ± 11.85	3.83 ± 0.75^a^	6.17 ± 0.70^a^	10.68 ± 1.99^a^
D	4.59 ± 0.35	0.06 ± 0.00^a^	40.64 ± 4.62	3.10 ± 0.74^a^	4.81 ± 0.27	9.46 ± 0.66^a^
E	3.93 ± 0.16	0.04 ± 0.00^a^	46.77 ± 8.78^a^	2.73 ± 0.32^a^	5.78 ± 0.67	9.50 ± 0.39^a^
F	3.46 ± 0.23	0.04 ± 0.00^a^	56.21 ± 5.43^a^	3.90 ± 0.63^a^	4.20 ± 0.56	9.48 ± 0.13^a^

*Note:* The values were expressed as mean ± SD of replicates.

Abbreviation: ComV, commercial variety (*V. vinifera*).

^a^Statistical grouping of the Dunnett test.

**Table 4:** Qualitative phytochemical analysis of *V. vinifera* and *A. indica.*

**Sample**	**Alkaloids**		**Flavonoids**	**Proanthocyanidins**	**Unsaturated sterols**	**Triterpenes**	**Phenolic compounds**	**Saponins**
	**Mayer's test**	**Wagner's test**	**Cyanidin test**	**Proanthocyanidin test**	**Liebermann–Burchard test**	**Salkowski test**	**Ferric chloride test**	**Froth test**
ComV	++	++	++	+	+	++	+++	ND
A	++	++	++	+	+	++	+++	ND
B	++	++	++	+	+	++	+++	ND
C	++	++	++	+	+	++	+++	ND
D	++	++	++	+	+	++	+++	ND
E	++	++	++	+	+	++	+++	ND
F	++	++	++	+	+	++	+++	ND

*Note:* The values were expressed as + = low, ++ = average, and +++ = high amount.

Abbreviations: ComV, commercial variety (*V. vinifera*); ND, not detected.

**Table 5:** Total flavonoid contents, total tannin contents, total phenolic contents, and antioxidant activities of *V. vinifera* and *A. indica* fruit samples.

**Sample**	**TFC (mg QE/100 g DW)**	**TTC (mg TAE/100 g DW)**	**TPC (mg GAE/100 g DW)**	**Antioxidant activity**		
				**DPPH IC**_**50**_ **(*μ*g/mL)**	**FRAP IC**_**50**_ **(*μ*g/mL)**	**ABTS IC**_**50**_ **(*μ*g/mL)**
ComV	3.50 ± 2.11^a^	0.18 ± 0.03^a^	21.17 ± 8.62^a^	24.26 ± 10.75^a^	52.30 ± 7.57^a^	58.20 ± 29.40^a^
A	1.36 ± 0.07^a^	0.26 ± 0.08^a^	11.48 ± 4.73^a^	63.30 ± 17.32	53.67 ± 7.80^a^	54.03 ± 17.25^a^
B	2.90 ± 0.79^a^	0.17 ± 0.02^a^	16.87 ± 4.77^a^	34.40 ± 8.74^a^	44.88 ± 6.20^a^	63.20 ± 34.60^a^
C	3.98 ± 0.12^a^	0.23 ± 0.04^a^	29.09 ± 6.05^a^	57.50 ± 24.20	50.65 ± 18.49^a^	58.60 ± 21.90^a^
D	2.47 ± 0.35^a^	0.25 ± 0.02^a^	25.17 ± 7.50^a^	17.38 ± 5.48^a^	66.50 ± 21.7^a^	62.65 ± 10.64^a^
E	4.09 ± 1.44^a^	0.13 ± 0.04^a^	27.72 ± 6.39^a^	35.31 ± 11.19^a^	48.35 ± 16.81^a^	64.40 ± 12.84^a^
F	4.23 ± 1.28^a^	0.22 ± 0.04^a^	25.24 ± 3.56^a^	33.27 ± 13.07^a^	61.63 ± 8.65^a^	79.60 ± 34.20^a^

*Note:* The values were expressed as mean ± SD of replicates.

Abbreviations: ComV, commercial variety (*V. vinifera*); mg GAE/100 g DW, milligrams of gallic acid equivalent/100 g dry weight of plant material; mg QE/100 g DW, milligrams of quercetin equivalent/100 g dry weight of plant material; mg TAE/100 g DW, milligrams of tannic acid equivalent/100 g dry weight of plant material.

^a^Statistical grouping of Dunnett test.

## Data Availability

The data presented in this study are available upon request from the corresponding author.
